# An Assessment of Potential Threats to Human Health from Algae Blooms in the Indian River Lagoon (USA) 2018–2021: Unique Patterns of Cytotoxicity Associated with Toxins

**DOI:** 10.3390/toxins15110664

**Published:** 2023-11-17

**Authors:** Esther A. Guzmán, Tara A. Peterson, Priscilla L. Winder, Kirstie T. Francis, Malcolm McFarland, Jill C. Roberts, Jennifer Sandle, Amy E. Wright

**Affiliations:** The Florida Center for Coastal and Human Health, Harbor Branch Oceanographic Institute, Florida Atlantic University, 5600 US 1 North, Fort Pierce, FL 34946, USA; tpitts3@fau.edu (T.A.P.); pwinder@fau.edu (P.L.W.); ktandberg2013@fau.edu (K.T.F.); mmcfarland@fau.edu (M.M.); jrober90@fau.edu (J.C.R.); jsandle@fau.edu (J.S.); awrigh33@fau.edu (A.E.W.)

**Keywords:** harmful algae blooms, phycotoxins, bioassays, cytotoxicity, metabolites

## Abstract

The Indian River Lagoon (IRL), a 156-mile-long estuary located on the eastern coast of Florida, experiences phytoplankton bloom events due to increased seasonal temperatures coupled with anthropogenic impacts. This study aimed to gather data on the toxicity to human cells and to identify secondary metabolites found in water samples collected in the IRL. Water samples from 20 sites of the IRL were collected during the wet and dry seasons over a three-year period. A panel of cell lines was used to test cytotoxicity. Hemagglutination, hemolysis, and inhibition of protein phosphatase 2A (PP2A) were also measured. Cytotoxic blooms were seen both in the south (*Microcystis*) and the north (*Pyrodinium*) of the IRL. Each toxin induced a consistent pattern of cytotoxicity in the panel of human cell lines assayed. During blooms, cytotoxicity due to a single type of toxin is obvious from this pattern. In the absence of blooms, the cytotoxicity seen reflected either a mixture of toxins or it was caused by an unidentified toxin. These observations suggest that other toxins with the potential to be harmful to human health may be present in the IRL. Moreover, the presence of toxins in the IRL is not always associated with blooms of known toxin-producing organisms.

## 1. Introduction

The Indian River Lagoon (IRL) is a 156-mile-long estuary located on the eastern coast of Florida, comprised of three lagoons: the Mosquito Lagoon, the Banana River, and the Indian River. The lagoon is where water from the Atlantic Ocean mixes with freshwater, resulting in brackish water that supports a wealth of biodiversity, from marsh snakes to fish and birds, as well as thousands of plants and animals [1]. In an assessment published in 2016, the East Central Florida Regional Planning Council (ECFPC) and the Treasure Coast Regional Planning Council (TCRPC) estimated that the IRL has an economic value of $7.6 billion dollars [2]. Recreation and tourism are an important part of this economic value, and it is estimated that by 2024, this region will receive about 11 million visitors annually [2]. The IRL borders five different counties and has five inlets that connect the lagoon with the Atlantic Ocean. One of these inlets in the northern IRL (Port Canaveral) has locks that limit the exchange of water [1], resulting in long residence times in this area of the lagoon.

The IRL ecosystem has been experiencing an increasing number of phytoplankton bloom events due in large part to increased seasonal temperatures coupled with anthropogenic impacts [3,4,5,6,7,8]. Other factors that contribute to increased blooms are the many shallow areas within the IRL and the fact that some areas have limited flushing and experience long water residence times [3]. Thus, an inflow of nutrients into the IRL and, in some areas, an inflow of fresh water are conditions that can lead to bloom formation. In a recently published assessment of the northern IRL (Mosquito Lagoon and Banana River) that covers a 23-year period from 1997 to 2020, it was noted that there is significant variability in bloom activity. Some of it is caused by cyclical patterns such as El Niño and La Niña events, while others by stochastic events such as tropical storms [3]. In the Southern Mosquito Lagoon, no blooms were seen between 2006 and 2010. In 2011, a picocyanobacterial bloom was observed. In 2012, a bloom by the brown tide alga *Aureoumbra lagunensis* that lasted from June to August was observed. Brown tide blooms were also seen in 2013 and 2015–2016. A nanoplankton bloom was reported in late 2019 and lasted until the winter of 2019, and a nonplanktonic *cyanobacterium* bloom was reported in the summer of 2020 [3]. In the Central Banana River, blooms of *P. bahamense,* picoplanktonic *cyanobacteria*, and diatoms were seen between 1997 and 2010. A bloom of pico-*Cyanobacteria* and nanoplanktonic eukaryotes was observed from March to October in 2011. In 2014, two diatom blooms were observed. In 2016, a brown tide bloom was observed in the winter and spring. The dissipation of this bloom in April was followed by a summer bloom of *P. bahamense,* and blooms of this algae were also seen in 2017 and 2020. A long-lasting bloom of *A. lagunensis* was observed starting in the winter of 2018 and lasting until the summer of 2019 [3]. This report also notices a regime shift with *A. lagunensis* and *P. bahamense* blooms becoming more common and containing larger amounts of biomass [3]. This regime shift coincided with a decrease in seagrasses and drift algae [3]. In the southern IRL, freshwater releases from Lake Okeechobee into the St. Lucie estuary have resulted in large blooms of the freshwater cyanobacterium *Microcystis aeruginosa*.

Algae blooms are known to produce a myriad of secondary metabolites, many of which can be toxic to humans [9,10]. This has led to the term harmful algal blooms (HABs). Collectively, the toxins produced by microalgae are known as phycotoxins [11]. Phycotoxins exhibit many different types of chemical structures: cyclic peptides (i.e., microcystin), amines (i.e., domoic acid), alkaloids (i.e., saxitoxins), and lipopolysaccharides, to name a few [11,12]. Phycotoxins can also be grouped by the target tissues affected: hepatotoxins, neurotoxins, cytotoxins, dermatoxins, and irritant toxins [12]. The most characterized and studied cyanobacterial toxin produced by HABs is microcystin LR, which is produced by *Cyanobacteria*. More than 90 structurally related microcystins have been reported in the literature [13]. Extensive blooms of *Microcystis* spp. caused by discharges from Lake Okeechobee into the St. Lucie Estuary have led to measurable amounts of microcystins in nasal swabs of people living and working near the area, but the health impacts are not yet defined [14]. Blooms by the brown tide alga *Aureoumbra lagunensis* are associated with fish kills, likely due to hypoxic conditions [15,16]. The blooms are associated with the growth of several pico- and nano-*Cyanobacteria* that have not been fully characterized taxonomically or for their biosynthetic abilities to produce toxins. *Pyrodinium bahamense* produces saxitoxin, which can accumulate in pufferfish in the IRL, leading to intoxication, which has led to the permanent closure of the pufferfish fishery in the IRL [17]. Blooms of the diatom *Pseudo-nitzschia* sp. are common in the IRL [3], and they are known to be able to produce domoic acid [18]. *Gambierdiscus toxicus* and *Karlodinium* sp. have also been observed in the IRL [3,19] and are known to be able to produce ciguatoxin [20] and hemolytic karlotoxins [21], respectively. The IRL also has a wealth of filamentous cyanobacteria, including *Moorea* sp. (formerly *Lyngbya* [22]). These occur both on the benthos as well as in large floating mats and are known to produce chemical compounds that deter animals from feeding on them and limit the growth of other organisms [23,24].

To increase our understanding of the environmental factors driving bloom and toxin production in the IRL, as well as the potential human health effects associated with toxins present in the IRL, the Harbor Branch Oceanographic Institute created the Florida Center for Coastal and Human Health. Our goal within the center was to gather data on the toxicity to human cells and suites of secondary metabolites (including known and new toxins) found in water samples collected in the IRL.

The first step towards this objective was to select a panel of cell lines to monitor the known effects of the phycotoxins. Microcystins present liver and neurological toxicity in humans, and these effects are directly associated with the expression of organic anion transporters (OATPs) in these tissues [13,25,26]. The OATPs are required to transport the microcystins into cells where they can exert their effects on protein phosphatases. To properly measure the cytotoxicity of the microcystins, it is essential to use cells that express OATPs to properly match the in vivo mechanisms of microcystin toxicity [26]. While certain non-small lung cell carcinomas [25] and pancreatic cancer cells [27] express high levels of OATPs, immortalized hepatocytes and hepatocyte cancer cells express low levels of these transporters [26]. Engineered lines that overexpress OATPs have been used to assess the cytotoxicity induced by microcystins as they better reflect in vivo activity [25,26]. Therefore, commercially available engineered human cell lines that overexpress OATPs (Corning™ TransportoCells, Corning, NY, USA) were used in this study.

Many phycotoxins are reported to be hepatotoxins, including microcystin MC, nodularin, cylindrospermopsin, pectenotoxins, and okadaic acid [12]. Hep G2 is a human hepatocellular carcinoma cell line that has been extensively used in the literature to understand liver function. This cell line retains many liver-specific functions and expresses many liver enzymes [28]. Okadaic acid has been reported to show potent cytotoxicity against this cell line [29], while microcystin LR exhibited minimal cytotoxicity [30], with low activity likely caused by the lack of OATPs [26]. Cylindrospermopsin has also been reported to exhibit cytotoxicity against this cell line [31]. This cell line has also been used to study phycotoxins present in mussels, such as saxitoxin, tetrodotoxin, domoic acid, and yessotoxin, with only the latter showing cytotoxicity against this cell line [32]. Many phycotoxins are reported to be neurotoxic, including microcystin LR, saxitoxins, β-methylamino-L-alanine (BMAA), brevetoxins, ciguatoxins, palytoxins, cyclic amines, and domoic acid [12]. The SH-SY5Y is a human neuroblastoma cell line that has been extensively used in the literature to understand neurotoxicity, as this cell line has many of the biochemical and functional properties of neurons [33]. SH-SY5Y is also considered a good model for neurotoxicity in vitro as these cells can be differentiated to have a mature neuronal phenotype through the addition of compounds to their media or simply by lowering the FBS content of the media [33]. This cell line has been used previously to understand the effects of BMAA [34], although an extremely high concentration of BMAA (2 mM) was required to see any cytotoxicity. Palytoxin has been reported to induce cytotoxicity in this cell line [35]. Maitotoxin, palytoxins, brevetoxins, and ciguatoxins are known to cause changes in membrane potential in this cell line [36]. Changes in membrane potential are associated with programmed cell death (apoptosis).

Exposure to some phycotoxins has been associated with renal failure [12]. The Vero cell line is a non-transformed cell line derived from the kidneys of African green monkeys. Cylindrospermopsin [31], anatoxin-a [37], and saxitoxin [38] have been shown to induce cytotoxicity in this cell line. Okadaic acid is known to inhibit protein synthesis in this cell line [39]. Some phycotoxins have effects on red blood cells. Karlotoxins are known to cause hemolysis [21] as membrane permeability is their mechanism of action [40]. Prymnesin [41] and palytoxin [42] are known to cause red blood cell lysis. Microcystins and anatoxin-a are known to cause hemagglutination [43]. Some phycotoxins are known to inhibit protein phosphatases (PP). Microcystin LR and okadaic acid inhibit PP1, PP2A, PP4, and PP5 with low nanomolar IC_50_s [44]. Nodularin also inhibits PP1, PP2A, and PP5 at low nanomolar concentrations [44]. Inhibition of protein phosphatases can be followed in vitro using commercially available kits.

Therefore, the suite of cell lines chosen for testing cytotoxicity in this study included OATP1 A2 and OATP1 B1 Transporto™ cells and Vero, Hep G2, and SH-SY5Y cells. In addition, hemagglutination and lysis were followed in sheep red blood cells, and the ability to inhibit protein phosphatase 2A (PP2A) was followed using an enzymatic commercially available kit. Samples that exhibited cytotoxicity, if available in sufficient quantity, were further subjected to liquid chromatography-high resolution mass spectrometry (LC-HRMS) analysis to assess the metabolites present in the sample.

## 2. Results and Discussion

Water samples from 20 sites of the IRL were collected during the wet and dry seasons over a three-year period; not all sites were collected in all years. This assessment was intended to determine if potential acute threats to human health were present in the IRL by measurement of cytotoxicity and related biological activities in extracts from water samples. Year 1 was collected in the fall (wet season) of 2018 and spring (dry season) of 2019. Data from year 1 were previously published in an assessment that focused on microcystins and saxitoxin [45]. Year 2 was collected in the fall of 2019 and spring of 2020. Year 3 was in the fall of 2020 and spring of 2021, and year 4 occurred in the fall of 2021. The disruption in the collection schedules was due to the COVID-19 pandemic. A map showing the locations of the collection sites is shown alongside the results.

Water samples were extracted, and these concentrated extracts were tested against the panel of cell lines described in the introduction to determine cell toxicity. Results were normalized against non-treated control and expressed as a percentage. Cytotoxicity is routinely measured in our laboratories for drug discovery. For this purpose, samples are tested at a concentration of 5 µg/mL, which usually leads to compounds active in the low micromolar range. For this study, samples were tested at a concentration of 10 µg/mL. This high concentration was used to detect as many metabolites as possible. Samples that present more than 50% cytotoxicity are considered active. The extraction process enriches organic components, and therefore, the concentration of cytotoxic compounds in the organic extracts is greater than the cytotoxicity that would be encountered if dealing with water samples that were not concentrated.

**Figure 1:**
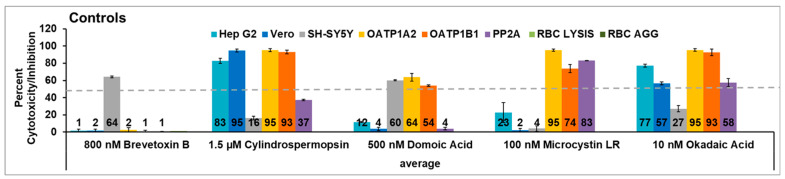
Effects of controls in cell line panel, red blood cells, and the enzyme protein phosphatase 2A. OATP1 A2 and OATP1 B1 Transporto™ cells and Vero, Hep G2, and SH-SY5Y cells were treated with 800 nM brevetoxin B, 1.5 µM cylindrospermopsin, 500 nM domoic acid, 100 nM microcystin LR, and 10 nM okadaic acid for 72 h. The results were normalized to solvent controls and expressed as a percentage. Those that induce more than 50% cytotoxicity (dotted line) are considered active. In addition, sheep red blood cells were treated with the same compounds for 4 h to measure hemagglutination or 6 h to measure cell lysis. None of the controls exhibited red blood cell lysis or hemagglutination at the concentrations tested. The effect of these compounds on protein phosphatase after 30 min of treatment is also shown. Unique patterns of cytotoxicity were seen with each of the known phycotoxins tested. The average of three independent experiments ± standard deviation is shown.

Control toxins were assayed concurrently with the extracts of the water samples. The concentrations used were based on the reported concentrations required to obtain fifty percent cytotoxicity (IC_50_) for the toxins in these cell lines. The controls used consisted of 800 nM brevetoxin B, 1.5 µM cylindrospermopsin, 500 nM domoic acid, 100 nM microcystin LR, and 10 nM okadaic acid. One finding of this study was that each toxin induced a consistent pattern of cytotoxicity in the panel of human cell lines assayed (Figure 1). Although unexpected, no effects were caused by the controls at these concentrations on red blood cell lysis or agglutination. The uniqueness of the pattern could be used to identify the presence of these toxins in the IRL samples and could serve as an identification tool for other samples assayed against the panel of cells used in this study.

**Figure 2:**
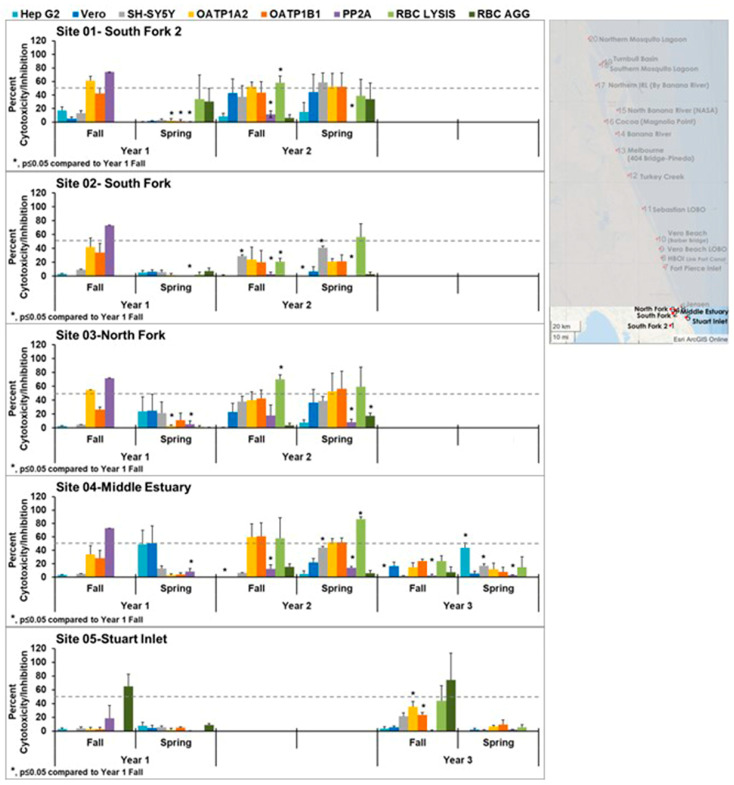
Effects of southernmost samples (sites 1–5) in cell line panel, red blood cells, and the enzyme protein phosphatase 2A. OATP1 A2 and OATP1 B1 Transporto™ cells and Vero, Hep G2, and SH-SY5Y cells were treated with 10 µg/mL samples for 72 h. Sheep red blood cells were treated with 50 µg/mL samples for 4 h to measure hemagglutination or 6 h to measure cell lysis. The effects of 10 µg/mL samples on protein phosphatase after 30 min of treatment are also shown. Those that induce more than 50% cytotoxicity (dotted line) are considered active. These samples exhibited cytotoxicity throughout all the sampling periods. During year 1, a *Microcystis* bloom occurred, and the pattern of cytotoxicity for microcystin LR is seen in sites 1–4. During years 2 and 3, cytotoxicity was observed but did not match any patterns, suggesting it was caused by either a mixture of toxins or an emerging toxin. The average of three samples ± standard error of the mean is shown. An asterisk denotes statistically significant differences (*p* ≤ 0.05) to fall year 1 data through Student’s *t*-test.

Three 1 L water samples were collected just below the surface per site. While there was enough reproducibility to see the same trend of patterns in each sample, the amount present differed enough to make the standard deviations substantial, and thus, the graphs for water samples show the standard error of the mean for the average of the three samples. For all assays, the height of the bar seems to correlate to the amount of toxin present, except for PP2A activity, as the linearity of this ELISA assay was limited. Statistically significant differences (*p* ≤ 0.05) to the fall 2018 levels are noted by an asterisk. Substantial seasonal variation was observed for samples collected in the southern IRL (sites 1–5; Figure 2).

Samples collected in fall 2018 (wet season) in the St Lucie Estuary (sites 1–4) showed cytotoxicity only against the OATP1 cell lines and inactivation of PP2A enzymatic activity in year 1. This pattern is consistent with the presence of microcystin LR and was confirmed by mass spectrometry and the observation of microcystins via ELISA in the water samples [45]. The transient nature of the bloom is seen by the fact that there are no traces of the bloom by the spring (dry) season, where little, if any, cytotoxicity was observed. Further evidence of the uniqueness of the pattern during the bloom is provided by the statistical analysis per season (Figure S1). The levels of cytotoxicity in the OATP1A2 cells in site 1 in fall year one were statistically different from all fall sites except sites 2–4, 6, 12, and 19. The cytotoxicity in OATP1B1 cells in fall year 1 site 1 was statistically different from all other fall sites except sites 2–4 and 6. The inhibition of PP2A activity from fall year 1 site 1 was statistically significantly different from all sites except sites 2, 5, 6, and 8. This seems to support the uniqueness of the pattern seen due to the *Microcystis* bloom in sites 1–4. These sites exhibited cytotoxicity in year 2, but the pattern did not clearly match any of the controls, suggesting the cytotoxicity was caused by either a combination of toxins or by an emerging toxin. Site 5 showed no presence of the bloom and little cytotoxicity in year 1. Some effects on red blood cell agglutination were seen at this site in years 1 and 3, but these were not statistically significantly different from other sites; thus, this site appeared to be the healthiest.

**Figure 3:**
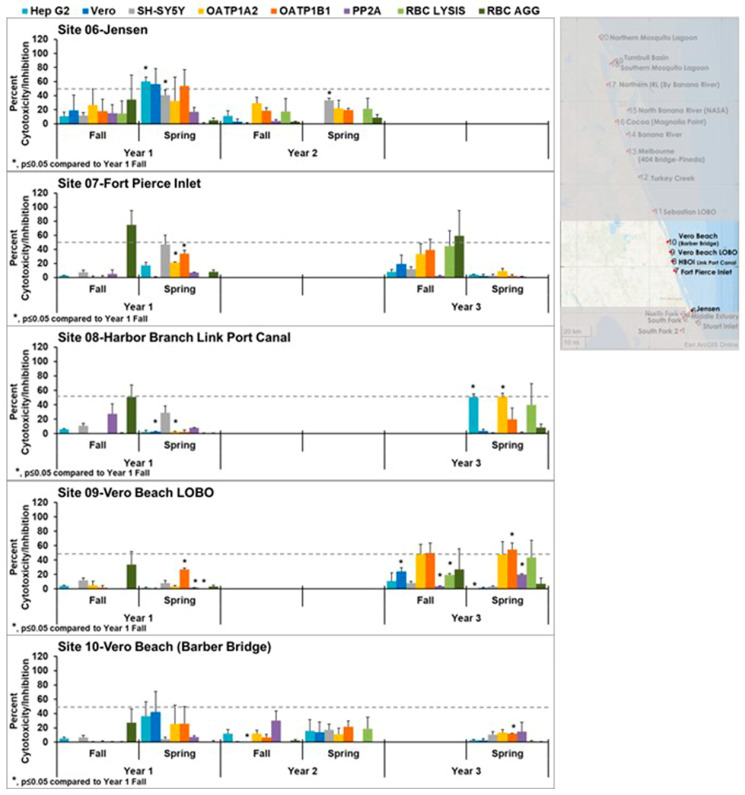
Effects of southern middle samples (sites 6–10) in cell line panel, red blood cells, and the enzyme protein phosphatase 2A. OATP1 A2 and OATP1 B1 Transporto™ cells and Vero, Hep G2, and SH-SY5Y cells were treated with 10 µg/mL samples for 72 h. Sheep red blood cells were treated with 50 µg/mL samples for 4 h to measure hemagglutination or 6 h to measure cell lysis. The effect of 10 µg/mL samples on protein phosphatase after 30 min of treatment is also shown. Those that induce more than 50% cytotoxicity (dotted line) are considered active. During year 1, site 6 (Jensen Beach) exhibited strong cytotoxicity. This was transient, as it was not seen at later times. Cytotoxicity observed in these sites did not match any patterns, suggesting it was caused by either a mixture of toxins or an emerging toxin. The average of three samples ± standard error of the mean is shown. An asterisk denotes statistically significant differences (*p* ≤ 0.05) to fall year 1 data through a Student’s *t*-test.

Figure 3 focuses on samples from sites 6–10. Samples from the Jensen Beach IRLON site (site 6) showed activity in all the cell lines tested in year 1, with a statistically significant increase in cytotoxicity in Hep G2 and SH-SY5Y between the fall and the spring. The cytotoxicity did not match the pattern of cytotoxicity of a single control, suggesting a mixture of toxins or an emerging toxin. This toxicity was transient as little activity was seen in year 2, with only the cytotoxicity in SH-SY5Y being statistically significantly different from that seen in the fall of year 1. Overall, sites 6–10 appear healthier as there were few samples with cytotoxicity above 50% in these sites, although there was statistically significant variation in these sites. Site 10 did show the pattern for cylindrospermopsin in the spring of year 1 but was not seen again at this site in years 2 and 3. There was some red blood cell agglutination in the fall of year 1 and year 3. This could be due to the influx of plant lectins, which are known to have this activity and could be introduced by run-off, which is common in these areas.

**Figure 4:**
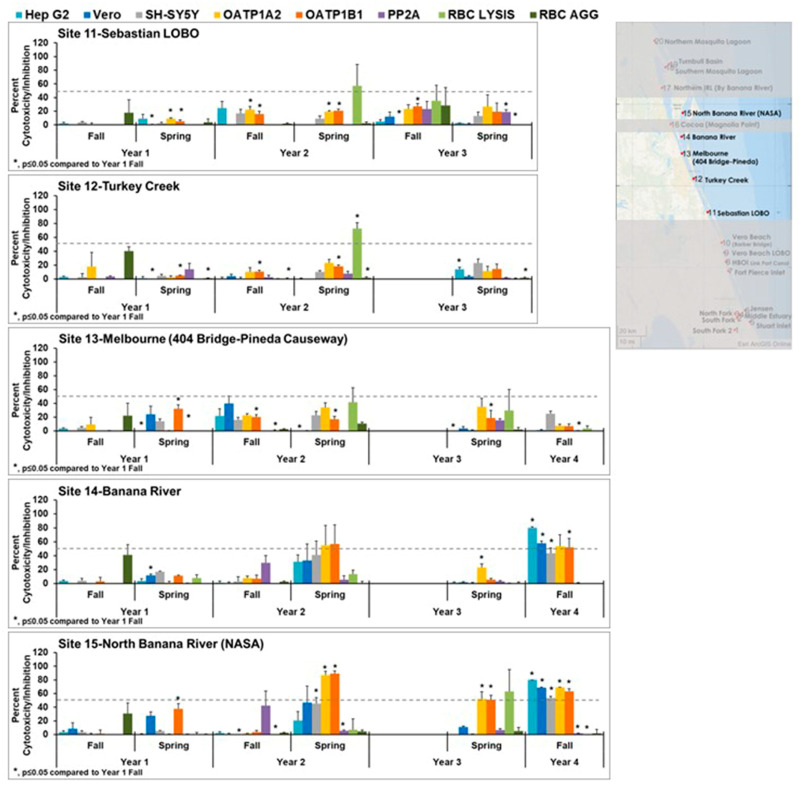
Effects of northern middle samples (sites 11–15) in cell line panel, red blood cells, and the enzyme protein phosphatase 2A. OATP1 A2 and OATP1 B1 Transporto™ cells and Vero, Hep G2, and SH-SY5Y cells were treated with 10 µg/mL samples for 72 h. Sheep red blood cells were treated with 50 µg/mL samples for 4 h to measure hemagglutination or 6 h to measure cell lysis. The effect of 10 µg/mL samples on protein phosphatase after 30 min of treatment is also shown. Those that induce more than 50% cytotoxicity (dotted line) are considered active. Overall, these appear to be healthier sites as less cytotoxicity was seen in these sites, except for sites 14 and 15, which exhibited some cytotoxicity in years 2 and 3. Cytotoxicity observed in these sites appears to match the pattern seen when testing samples from a *Pyrodinium* bloom. The average of three samples ± standard error of the mean is shown. An asterisk denotes statistically significant differences (*p* ≤ 0.05) to Fall year 1 data through a Student’s *t*-test.

Moving north on the IRL, Figure 4 focuses on sites 11–15. Overall, these sites exhibited less cytotoxicity than the sites to the south. Sites 14 and 15 showed some cytotoxicity during the spring of year 2 and fall of year 4. The cytotoxicity seen in site 14 in the fall of year 4 was statistically significantly different from fall of year 1. The cytotoxicity seen in site 15 in the spring year 2, spring year 3, and fall year 4 were statistically different from fall year 1. While the patterns did not match the controls, the patterns of cytotoxicity are consistent between these two sites within the collection times, suggesting the same toxin or mixture of toxins is present.

**Figure 5:**
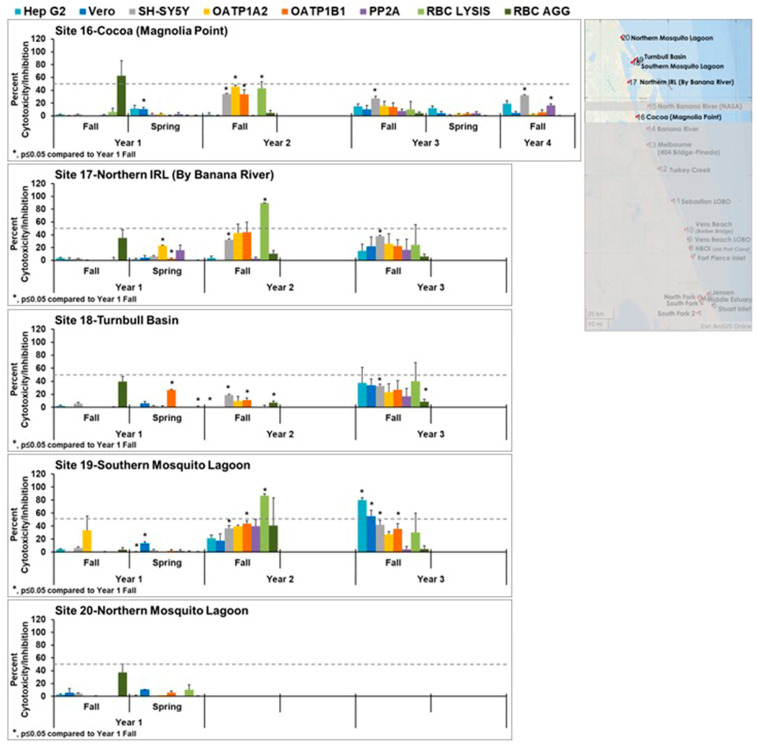
Effects of northern samples (sites 16–20) in cell line panel, red blood cells, and the enzyme protein phosphatase 2A. OATP1 A2 and OATP1 B1 Transporto™ cells and Vero, Hep G2, and SH-SY5Y cells were treated with 10 µg/mL samples for 72 h. Sheep red blood cells were treated with 50 µg/mL samples for 4 h to measure hemagglutination or 6 h to measure cell lysis. The effects of 10 µg/mL samples on protein phosphatase after 30 min of treatment are also shown. Those that induced more than 50% cytotoxicity (dotted line) are considered active. Overall, these appear to be healthier sites as less cytotoxicity was seen in these sites, except for sites 17 and 19, which exhibited some cytotoxicity in years 2 and 3. Cytotoxicity observed in these sites did not match any patterns, suggesting it was caused by either a mixture of toxins or an emerging toxin. The average of three samples ± standard error of the mean is shown. An asterisk denotes statistically significant differences (*p* ≤ 0.05) to fall year 1 data through a Student’s *t*-test.

The cytotoxicity for the most northern sites 16–20 is shown in Figure 5. Again, these sites exhibited less cytotoxicity than the sites to the south. In some year 2 wet (fall) and dry (spring) season samples and year 3 wet (fall) season samples, red blood cell lysis was observed and was statistically significantly different from year 1. This was not observed in year 1. This lysis could be caused by the introduction of plant saponins, which are known to have this activity. Those samples that exhibited cytotoxicity did not have patterns that matched the controls, suggesting the presence of mixtures or different toxins.

**Figure 6:**
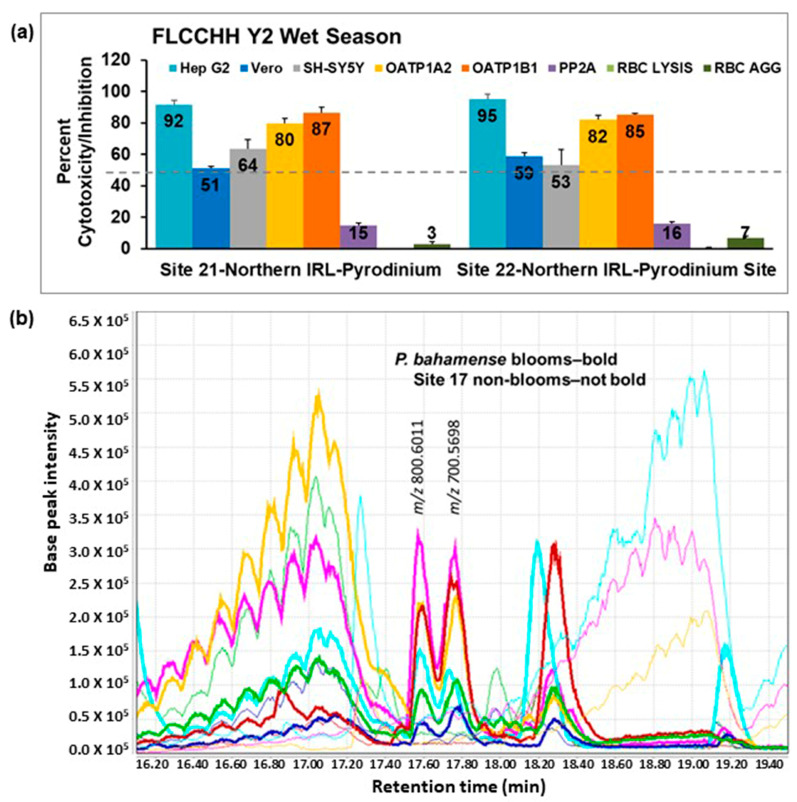
(**a**) Cytotoxicity patterns of samples collected during a *Pyrodinium* bloom. OATP1 A2 and OATP1 B1 Transporto™ cells and Vero, Hep G2, and SH-SY5Y cells were treated with 10 µg/mL samples for 72 h. Sheep red blood cells were treated with 50 µg/mL samples for 4 h to measure hemagglutination or 6 h to measure cell lysis. The effects of 10 µg/mL samples on protein phosphatase after 30 min of treatment are also shown. Those that induced more than 50% cytotoxicity (dotted line) are considered active. A consistent pattern of cytotoxicity was seen in these samples. The average of three samples ± standard deviation is shown. (**b**) Comparison of LC-HRMS chromatograms of cytotoxic bloom and non-cytotoxic, non-bloom samples from the NIRL sites. The compounds at *m*/*z* 800.6011 and 700.5698 are only found in cytotoxic bloom samples and were originally proposed to be responsible for the observed cytotoxicity. Further separation by preparative HPLC led to the observation that the cytotoxic compound is a very minor component of the extract. The compounds with *m*/*z* 800.6011 and 700.5698 are present in all bloom samples from these sites and may have other roles as allelochemicals.

During this period, in addition to the *Microcystis* bloom in year one, there was a *Pyrodinium* bloom in year 2, shown in Figure 6a. Samples from this bloom show a consistent pattern of cytotoxicity, suggesting that the same toxin may be responsible. LC-HRMS data for these cytotoxic extracts and inactive drcy season extracts from the same location were compared using MzMine 2.53 and suggested the presence of two compounds (“putative toxins”, *m*/*z* 700.5698 and 800.6011) which co-occur in the cytotoxic samples but not in the non-bloom inactive samples, Figure 6b. Preparative HPLC on one of the active extracts from a bloom site was conducted using a Sunfire C18 prep column and a similar gradient to that used in the LC-HRMS analysis (H_2_O-CH_3_CN containing 0.1% formic acid) to provide a series of fractions which were submitted for bioassay. Surprisingly, none of the fractions were active, suggesting that the active compound could be unstable to acid. The same gradient and column were used to separate another aliquot of the extract but without the formic acid modifier. In this case, a fraction that eluted immediately after the solvent front was cytotoxic. Analysis of the cytotoxic fraction by LC-HRMS indicated that the two “putative toxins” were present in this fraction, consistent with our hypothesis that the active compounds may be acid-labile. Saxitoxin was not detected in the cytotoxic fraction and would not be expected to be acid-labile. A separation using an amino-functionalized stationary phase was developed to optimize the purification of the two “putative toxins”, but the cytotoxicity did not correlate to these compounds and was rather associated with an extremely minor component present in the fraction. Further work is required to purify sufficient material to conduct structure elucidation of the toxic compound present in these *Pyrodinium* bloom samples. The compounds originally proposed as putative toxins were consistently observed in the *Pyrodinium* bloom samples and may have allelochemical or other activity within the bloom.

**Figure 7:**
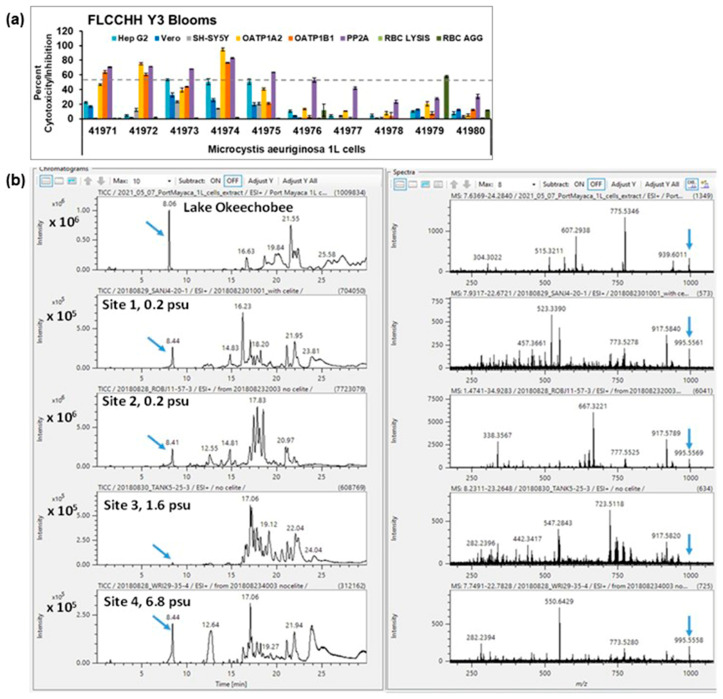
(**a**) Cytotoxicity patterns of a sample collected during a *Microcystis* bloom in Lake Okeechobee. OATP1 A2 and OATP1 B1 Transporto™ cells and Vero, Hep G2, and SH-SY5Y cells were treated with 10 µg/mL samples for 72 h. Sheep red blood cells were treated with 50 µg/mL samples for 4 h to measure hemagglutination or 6 h to measure cell lysis. The effects of 10 µg/mL samples on protein phosphatase after 30 min of treatment are also shown. Those that induced more than 50% cytotoxicity (dotted line) are considered active. Sample 41971 is the crude extract, and the other samples shown are fractions derived from the crude extract, with 41972 and 41974 containing microcystins. Sample 41974 is highly enriched in microcystin LR and exhibits the same pattern of cytotoxicity as authentic microcystin LR control. The average of two replicates ± standard deviation is shown. (**b**) Comparison of LC-HRMS chromatograms for samples at 4 sites in the St Lucie estuary and SIRL during the 2018 *Microcystis* bloom and from Lake Okeechobee 2021. Blue arrows designate the peak for microcystin LR (MLR). The lake and site 2 had the highest concentrations of microcystin LR.

In 2021, samples were collected from a *Microcystis* bloom occurring lakeside at the Port Mayaca lock in Lake Okeechobee. Water from Lake Okeechobee is released through the lock into the C-44 canal and ultimately flows into the St Lucie Estuary and southern IRL and is the likely source of most *Microcystis* blooms in the southern IRL. The pattern of cytotoxicity from microcystin LR was seen in the crude extract (41941 in Figure 7a). Fractionation of the crude extract yielded two fractions (41972 and 41974, Figure 7a), which appear by ^1^H NMR to be highly enriched in microcystins, with 41974 being microcystin LR based upon mass spectrometric analysis. An amount of 2 mg of enriched microcystins was purified from 1 L of water. These samples collected during blooms support our observations that toxins have a unique pattern of cytotoxicity in our panel of cell lines.

## 3. Conclusions

Cytotoxic blooms were seen both in the south (*Microcystis*) and the north (*Pyrodinium*) of the IRL. In the absence of blooms, sites 1–4 in the south IRL and sites 14 and 15 in the north IRL appeared to have the most cytotoxicity during the time of the assessment.

An important finding of this study was that each toxin induced a consistent pattern of cytotoxicity in the panel of human cell lines assayed. The uniqueness of the pattern could be used to identify the presence of these toxins in the IRL samples and could serve as an identification tool for other samples assayed against the panel of cells used in this study. This was exemplified when collecting samples during blooms, as a consistent pattern of cytotoxicity was seen in similar samples. Using an assay panel to assess the presence of toxic materials could allow for better monitoring of human health impacts, especially from emerging toxins within the system.

The pattern of cytotoxicity that appears to correlate with microcystins was only seen in year 1 when there was a *Microcystis* bloom happening during the fall (wet season) of that year in the southern IRL (sites 1–4). The pattern was not seen in the dry season samples from that year, which highlights the temporal appearance of the bloom. The pattern was not seen again until testing water samples from a bloom of *Microcystis* in Lake Okeechobee in year 3. These observations suggest that the cytotoxic effects of microcystins are only measurable in the IRL when there is an active bloom. If microcystins were present at any other time, their concentration was not sufficient to induce cytotoxicity in the cells used. Moreover, microcystin LR only shows cytotoxicity in cells with active organic anion transport. Overall, it appears that microcystins are primarily a threat to human health in the IRL during blooms, and because of the necessity of active transport, the toxin would need to be ingested or inhaled to present a threat to human health. Ingestion can be avoided by filtering water through activated charcoal [46]. Similarly, effects due to inhalation are effectively blocked by the mucus membrane, which traps toxins that are subsequently eliminated through coughing [47]. However, pet and wildlife exposures can still occur.

A major question we sought to answer through this study was whether there are emerging toxins or other signaling molecules associated with HABs in the IRL. The data collected to date suggest that this is indeed the case. One of the major findings of this assessment was that each toxin used as a control gives a unique pattern of cytotoxicity that could be used to determine the presence of the toxin in environmental samples. During blooms, cytotoxicity due to a single type of toxin is obvious from this pattern. In the absence of blooms, when cytotoxicity was seen, the pattern looked more like it was due to a mixture of toxins or was caused by an emerging toxin. These observations suggest that other toxins with the potential to be harmful to human health may be present in the IRL. Moreover, the presence of toxins in the IRL is not always associated with blooms of known toxin-producing organisms. One factor that hampers the identification of these new metabolites is the low yield from the one-liter samples of water. Additional research needs to be performed to determine what is causing this cytotoxicity. The use of methods such as tangential flow filtration to allow for the extraction of larger volumes of water and increasing the number of cells sampled may allow for the identification of the active compounds. Further work also needs to be carried out to better understand the role of other non-toxic compounds consistently observed in the bloom samples.

## 4. Materials and Methods

Reagents. The 3-[4,5-Dimethyl-2-thiazolyl]-2,5-diphenyl-2H-tetrazolium bromide (MTT) used for cell viability assays was purchased from Millipore Sigma (St. Louis, MO, USA). Cylindrospermopsin was obtained from Enzo Life Sciences (Farmingdale, NY, USA), brevetoxin B was obtained from Cayman Chemical (Ann Arbor, MI, USA), microcystin LR was obtained from EMD (now Millipore Sigma, Burlington, MA, USA), and okadaic and domoic acid were obtained from Millipore Sigma. Supelco Diaion^®^ HP-20 was purchased from Sigma-Aldrich (St. Louis, MO, USA)

Cell Culture. The human hepatocellular carcinoma Hep G2 (ATCC^®^ HB-8065), the human neuroblastoma SH-SY5Y (ATCC^®^ CRL-2266), and the “normal” African Green Monkey Vero (ATCC^®^ CCL-81) cell lines were obtained from the American Type Culture Collection (ATCC, Manassas, VA, USA). The engineered TransportoCells™ OATP1A2 and OATP1B1 were purchased from Corning (Corning, NY, USA). Hep G2 cells were cultured in F12K Medium (N3520, Sigma, St. Louis MO, USA), supplemented with 10% FBS, 1X antibiotic/antimycotic (Gibco 15240-096), and 50 µg/mL gentamicin (Gibco 1570-060). OATP Cells were cultured in DMEM medium (ATCC 30-2002) supplemented with 10% FBS (not heat inactivated), 18 mM HEPES, 1X antibiotic/antimycotic (Gibco 15240-096), and 50 µg/mL gentamicin (Gibco 1570-060). SH-SY5Y were cultured in RPMI 1640 Medium supplemented with 10% FBS, 2 mM L-glutamine, 18 mM HEPES, 1X antibiotic/antimycotic (Gibco 15240-096), and 50 µg/mL gentamicin (Gibco 1570-060). Vero cells were cultured in RPMI 1640 Medium supplemented with 10% FBS, 55 mg sodium pyruvate, 4.5 g/L D-glucose, 2 mM L-glutamine, 18 mM HEPES, 1X antibiotic/antimycotic (Gibco 15240-096), and 50 µg/mL gentamicin (Gibco 1570-060).

Extraction. Three replicate water samples (1 L) were collected at each of the 20 sites during the wet (fall) and dry (spring) seasons over a three-year period [45]. The primary goal was to provide organic extracts for biological testing and for liquid chromatography-high resolution mass spectrometry (LC-HRMS) analysis. Over the course of the project, different extraction methods were used in an attempt to minimize the detection of contaminants such as polyethylene glycol (PEG) and maximize the detection of organic compounds present in the samples. Notably, when blooms were sampled, the extract yields were higher than for samples where no bloom was reported, and PEG contamination was minimally observed in bloom samples. This was especially true for the *Microcystis* bloom, where a simple filtration of cells through filter paper gave good extracts with no contaminants observed. For samples where no obvious blooms were detected, the yields were extremely low (0.5 mg or less), and the LC-HRMS analysis was often dominated by contaminants (primarily multiple forms of polyethylene glycol, (PEG)) which overwhelmed the LC-HRMS analysis. On the mass spectrometer used in this study, PEG gives a very strong signal, which can obscure minor components. Many of the PEG contaminants could be traced to the filter paper or the resin used in our various extraction processes, although the *m/z* observed for some PEGs did not match these known contaminants and are most likely from the water samples themselves. PEGs are ubiquitous in sunscreens, cosmetics, personal care products, industrial chemicals, and medicines. In some cases, to allow for direct comparison of extracts from the same site, the original extraction method was used, recognizing that some PEG contamination could be introduced. Methods used for each sample can be found in Table S1.

Method 1: Extracts were prepared by filtering 1 L of water (3 replicates) collected at the surface through Whatman Number 3 filter paper and then extracting the filter with CH_3_OH (3 × 100 mL) followed by a mixture of CH_3_COOCH_2_CH_3_ in CH_3_OH (1:1 *v*/*v*, 1 × 100 mL). The extracts were filtered and concentrated to dryness under reduced pressure to yield a residue. The dried residue was desalted by the addition of 2 mL of H_2_O and 2 mL of CH_3_COOCH_2_CH_3_, followed by placing the sample into a −20 °C freezer to freeze the water phase. Then, the CH_3_COOCH_2_CH_3_ phase was removed by pipette and dried under a stream of nitrogen to yield the final extract used for testing.

Method 2: Extracts were prepared as follows: 1 L water samples collected at the surface (3 replicates) were sonicated using a sonic dismembrator (Fisher Scientific Model 500) for 2 min to lyse cells, then 2 g of washed Diaion^®^ HP-20 resin was added. The samples were frozen overnight and then allowed to come to room temperature the following day. The water sample was then filtered using a glass Büchner funnel fitted with Whatman No. 3 filter paper, and the resin and filter were washed with methanol (3 × 100 mL) followed by CH_3_OH:(CH3)_2_CHOH (1:1 *v*/*v*, 1 × 100 mL). The combined washes were concentrated to dryness under reduced pressure. The samples were then desalted as described above.

Method 3: Extracts were prepared as follows: 1 L water samples collected at the surface (3 replicates) were sonicated using a sonic dismembrator for 2 min to lyse cells, then 2 g of washed Diaion^®^ HP-20 resin was added. The samples were frozen overnight and then allowed to come to room temperature the following day. The water sample was then filtered using a fritted glass funnel (no filter paper), and the resin and filter were washed with CH_3_OH (3 × 100 mL) followed by CH_3_OH:(CH3)_2_CHOH (1:1 *v/v*, 1 × 100 mL). The combined washes were concentrated to dryness under reduced pressure. The samples were then desalted as described above.

Method 4: Extracts were prepared as follows: 1 L water samples collected at the surface (3 replicates) were sonicated using a sonic dismembrator for 2 min to lyse cells, then frozen overnight. After thawing, the samples were partitioned with *n*-butanol (3 × 100 mL). The organic phase was dried under reduced pressure and then desalted as described above.

Cytotoxicity Assay (MTT). Cells were plated on a 384-well tissue culture plate at a volume of 30 μL/well. The number of cells/well used was as follows: 1500 for Vero, 6000 for Hep G2 and SH-SY5Y, and 3000 for OATP cells. Cells were allowed to adhere overnight. At the end of this incubation, 30 μL of medium containing treatment was added, consisting of 10 μg/mL of the samples, media alone, or media with methanol (solvent control). Pure phycotoxins, including 1.6 µM brevetoxin, 10 nM okadaic acid, 100 nM microcystin LR, 1.5 µM cylindrospermopsin, and 500 nM domoic acid, were tested at the same time to serve as controls. The cells were incubated for 72 h at 37 °C and 5% CO_2_. After this incubation, 25 μL of a 5 mg/mL solution of MTT were added to each well. Cells were incubated for 3 h at 37 °C. After centrifugation and removal of the supernatant, 100 μL of acidified isopropyl alcohol (1:500 hydrochloric acid to isopropanol solution) was added to each well. The crystals were dissolved by shaking or pipetting. The absorbency of each well was measured at 570 nm with a plate reader (NOVOstar, BMG Labtech Inc., Durham, NC). The resulting absorbencies were normalized against the solvent control and plotted using Microsoft Excel (Microsoft Office 365).

Hemagglutination Assay. This assay was adapted from a previously published protocol by Carmichael, W. W et al. [43]. Sheep red blood cells (erythrocytes) were purchased from Innovative Research (ISHRBC100P15ML; Novi, MI, USA). Erythrocytes were diluted 1:50 in PBS. An amount of 20 µL of diluted cells was plated into a 96-well U-bottom clear plate. Treatments were pipetted on top of cells at a volume of 20 µL per well. Treatments consisted of 50 μg/mL water samples, solvent controls, phycotoxin controls, 25 µg/mL anti-sheep red blood cell antibody (assay control, 213–4139, Rockland Immunochemicals, Pottstown, PA, USA), and 50 µg/mL concanavalin A (positive control; C0412, Sigma, St. Louis MO, USA). Samples were tested in duplicate. Cells were incubated with treatments for 3 to 4 h at room temperature. At the end of this incubation, the plate was imaged with ChemiDoc MP (Bio-Rad, Hercules, CA, USA), using the single channel protocol, gel imaging, and Cy3 blots application. Image exposure was optimized automatically by the software for faint bands. Image was analyzed with Image Lab Software 6.1 (Bio-Rad, Hercules, CA, USA) using the volume tools. The resulting volumes were exported to Microsoft Excel (Microsoft Office 365), and the results from the duplicate were averaged and normalized to the antibody control, which represents 100% agglutination.

Red Blood Cell Lysis. Adapted from previously published protocols by Malagoli, D. et al. [48]. Samples for testing were diluted to a testing concentration of 50 µg/mL in a 10 µL volume of PBS and plated onto a 384-well V bottom clear plate. Sheep red blood cells were diluted 1:50 with PBS, and then 90 µL was plated on top of treatments. A 20% Triton X-100 solution was used as the assay control, and PBS was used as the negative control. The natural compound saponin (20 µg/mL) was used as a positive control. Plates were incubated at 37 °C for six hours as this was the optimal time for monitoring lysis caused by palytoxin. At the end of the incubation, plates were centrifuged to pellet intact erythrocytes. A total of 50 µL supernatant was transferred into a clear, flat-bottom 384-well plate, and absorbance was read at 405 nm wavelength using the plate reader (NOVOstar, BMG Labtech Inc., Durham, NC, USA). The average of the absorbance readings for the negative control samples was subtracted from all other samples’ averages. The resulting absorbance was normalized against the assay control, which represents 100% hemolysis and is expressed as a percentage.

Protein Phosphatase 2A (PP2A) Assay. This assay used the Eurofins/Abraxis Kit for Microcystins/Nodularins PP2A (520032, Warminster, PA, USA) and was performed according to manufacturer’s instructions, except for adapting it to be used on a 384-well plate. All reagents were allowed to reach room temperature (23 ± 3 °C) before starting the assay. To prepare phosphatase solution, 3 mL of Phosphatase Dilution Buffer was added to one of the Phosphatase vials and mixed carefully by inversion. The solution was gently shaken at room temperature for 1 h to ensure that the enzyme was fully hydrated. Reaction controls include complete reaction, reaction with no substrate, and reaction with no enzyme. The kit includes four microcystin LR standards at concentrations of 0.25, 0.5, 1, and 2.5 nM. Water samples and solvent controls were tested at 10 µg/mL. Assay controls included kit standards, 10 mM Na_3_VO_4_, 10 nM okadaic acid, and 100 nM microcystin LR. Samples were plated at a volume of 5 μL/well in duplicate, followed by the addition of 7 μL of the Phosphatase Solution to each well. This was followed by the addition of 9 μL of Chromogenic Substrate to each well. The plate was covered with adhesive film and incubated for 30 min at 37 °C. At the end of this incubation, 7 μL of Stop Solution was added to each well, and the absorbance of samples was read at 405 nm wavelength using the plate reader (NOVOstar, BMG Labtech Inc., Durham, NC, USA). Average absorbance from test samples was normalized against the average absorbance for the complete reaction, which represents 100% activity using Microsoft Excel (Microsoft Office 365).

Secondary Metabolite/Toxin Profiling. LC-HRMS analysis was conducted in positive ion detection mode using a JEOL Accu-TOF (JEOL USA, Peabody, MA, USA) connected to an Infinity II HPLC system (Agilent Corporation, Santa Clara, CA, USA). LC-HRMS analyses were completed using a reversed-phase HPLC column (Waters Sunfire C18, 5 μm, 3.0 mm × 100 mm, Waters Corporation, Milford, MA, USA). Each sample was analyzed at a concentration of 1.0 mg/mL in CH_3_OH with a 5 μL injection. An internal standard of 25 ng reserpine was added to each sample. Chromatographic separation was accomplished using the following conditions: Solvent A: H_2_O containing 0.1% formic acid and Solvent B: CH_3_CN containing 0.1% formic acid; t = 0 min 80:20 A:B *v*/*v*; t = 14 min 100% B; t = 29 min 100% B t = 30 min 80:20 A:B *v*/*v*, followed by 6 min re-equilibration, flow rate = 0.3 mL/min. Mass analysis in positive ion mode was completed over a *m*/*z* range of 150–2000 amu. The settings were set as follows: needle voltage 2000 V, desolvating chamber temperature 250 °C, detector 2300 V, orifice 1 85 V and 80 °C, ring lens, 15 V, orifice 2 5 V, ion guide RF 1500 V, ion guide bias 27 V, focus 10 V, quad 20 V, left/right 0 V, top/bottom 0.6 V, focus lens −120 V, push bias 0.36 V, reflectron 990 V, push 778 V, pull −778 V, and flight tube −7000 V.

Fractionation of the extract from *Pyrodinium* bloom (year 2, site 21).

First preparative HPLC separation on C-18 with formic acid. A 1 L sample collected during a *Pyridinium* bloom at site 22 in the northern IRL was extracted per the normal procedure, yielding 8 mg of extract. This material was separated by preparative HPLC on a Waters Sunfire C18 OBD 10 µm column (150 mm × 19 mm) using the following conditions: Solvent A: H_2_O +0.1% formic acid and Solvent B: CH_3_CN + 0.1% formic acid; t = 0 min A:B 90:10 *v*/*v*, t = 20 min 100%B; t = 28 min 100% B; t = 30 min A:B 50:50; flow rate =12 mL/min; detection photodiode array (PDA) and ESI MS (positive and negative mode); and carrier solvent CH_3_CN with 0.1% formic acid. Samples were collected by time, with a total of 8 fractions collected. No fractions showed activity against the Vero, Hep G2, OATP1A2, or OATP1B1 cell lines.

Second preparative HPLC on C-18 without formic acid. The extract used in the above experiment was separated by preparative HPLC on a Waters Sunfire C18 OBD 10 µm column (150 mm × 19 mm) using the following conditions: Solvent A: H_2_O:CH_3_CN 95:5 and Solvent B: CH_3_CN; t = 0 min A:B 90:10 *v*/*v*, t = 20 min 100% B; t = 28 min 100% B; t = 30 min A:B 50:50 *v/v*; flow rate =12 mL/min; detection by PDA and ESI MS (positive and negative mode); and carrier solvent CH_3_CN with 0.1% formic acid. Samples were collected by time, with a total of 6 fractions collected. The fraction eluting at the solvent front showed 51% inhibition of the Vero Cell line, 94% inhibition of the Hep G2 cell line, 84% inhibition of the OATP1 A2 cell line, and 57% inhibition of the OATP1 B1 cell line.

Third preparative HPLC separation. A 4 L sample (9-VIII-2019-21-004) collected from the NIRL was sonicated using a sonic dismembrator for 2 min to lyse cells, and then approximately 4 g of Diaion^®^ HP-20 resin was added. The sample was frozen overnight, thawed the following day, and then filtered through a fritted glassfunnel. The retained resin was washed with 2 × 400 mL portions of CH_3_OH followed by 1 × 400 mL portion of CH_3_OH:(CH3)_2_CHOH 3:1 *v*/*v*. The combined extracts were concentrated by distillation under reduced pressure to yield 189 mg of extract. This extract was desalted by partitioning between H_2_O (1 mL) and CH_3_COOCH_2_CH_3_:CH_3_OH (9:1 *v*/*v*) (2 mL), 4 ×. This material was dried down under a stream of N_2_ and after reconstitution was separated by preparative HPLC on a Phenomenex Luna 5µ NH_2_ 100 Å Axia packed column (100 × 21.2 mm) using the following conditions: Solvent A: H_2_O:CH_3_CN 95:5 *v/v*, Solvent B: CH_3_CN; t = 0 min 100% B, t = 20 min 100% A; t = 22 min 100% A; t = 23 min A:B 50:50 *v*/*v*; flow rate =12 mL/min; detection by PDA and ESI MS (positive and negative mode); and carrier solvent CH_3_CN with 0.1% formic acid. Samples were collected by time, with a total of 7 fractions collected. Fraction 5 (WIN22-77-5), which eluted between 8 and 11 min, showed 78% inhibition against the Vero cell line at 10 µg/mL but had no activity against the other cell lines.

Extraction and chromatography of Port Mayaca Lock *Microcystis* bloom. A 1 L water sample was collected at the surface during a bloom of *Microcystis* on the lakeside immediately north of the Port Mayaca Lock, Lake Okeechobee. The sample had a dense number of cyanobacterial cells floating on the surface. The sample was filtered using a porcelain Büchner funnel (~4” diameter) fitted with two pieces of Whatman #2 filter paper. The filter paper was wetted with LC-MS grade methanol, and the methanol was discarded. (Note that the water filtrate after this filtration process was a very light brown; it did not have any obvious large *Microcystis* cells or cell aggregations.) The filter, which contained the cells, was placed into a beaker containing approximately 50 mL of LC-MS grade CH_3_OH and steeped at −20 °C. The methanol extract of the cells was decanted and filtered using a Büchner funnel and Whatman #3 paper. The filter paper and cells from the original filtration were macerated with a spatula and extracted exhaustively with CH_3_OH followed by CH_3_OH:CH_3_COOCH_2_CH_3_ 1:1. The combined extracts were dried by distillation under reduced pressure and then transferred to a 20 mL scintillation vial and dried under a stream of N_2_. The total yield of extract was 0.0876 g. A total of 2 mg was subsampled to make a 1 mg/mL solution for LC-HRMS analysis, and the remaining extract was fractionated by medium-pressure liquid chromatography (MPLC) on an Isco Combi-Flash Rf_4x_ (Teledyne ISCO, Lincoln, NE, USA) using a 5.5 g Redi-Sep C18 column and a linear gradient of CH_3_CN and H_2_O followed by washes with CH_3_OH, CH_2_Cl_2_, and a CH_3_CN:H_2_O:CF_3_COOH (50:50:0.1 *v/v/* as follows: Solvent A: H_2_O, Solvent B: CH_3_CN, Solvent C: CH_3_OH, Solvent D: CH_2_Cl_2_); 0 CV A:B (94:6 *v/v*) hold for 5.2 CV; then linear gradient to 100% B over 40.8 CV and hold for 7.1 CV at 100% B; switch to 100% C hold for 3.9 CV; linear gradient to 100% D over 7.1 CV, hold for 2 CV at 100% D, wash with 100% C for 5.3 CV, and then wash with CH_3_CN:H_2_O:CF_3_COOH (50:50:0.1 *v/v/v*) for 5 CV. Fractions were collected as follows: 41972: 0–9 CV; 41973 9–20 CV; 41974 20–30 CV; 41975 30–42 CV; 41976 42–60 CV; 41977 60–66 CV; 41978 66–68 CV; and 41979 68–75 CV; 41980 TFA wash. Biological activity and ^1^H NMR data consistent with that expected for microcystins were observed in 41972 and 41974.

Statistical Analysis: Student’s *t*-test was calculated per site by comparing the data of later samplings against the fall 2018 (year 1) data using Microsoft Excel (Microsoft Office 365). A *p* value less than or equal to 0.05 was considered significant. Student’s *t*-test was also used to compare the data per season for each year again using the year 1 data as basal levels, which is shown in Figure S1.

## Data Availability

Raw Data for each water sample, as well as the extraction method, are listed in Table S1. Any data not included in the manuscript or supplementary materials that support the work presented in this manuscript are available upon reasonable request to the corresponding author (E.A.G.).

## Supplementary Materials

The following supporting information can be downloaded at https://www.mdpi.com/article/10.3390/toxins15110664/s1. Table S1: Raw Data from cytotoxicity assays, red blood cell assays, PP2A, and method for extraction; Figure S1: Statistical analysis of the data by season per year.
