# Supplementary figures and images for: An Assessment of Potential Threats to Human Health from Algae Blooms in the Indian River Lagoon (USA) 2018–2021: Unique Patterns of Cytotoxicity Associated with Toxins
Source: Toxins (Basel). 2023 Nov 17;15(11):664. doi: 10.3390/toxins15110664 (PMC10675324; doi:10.3390/toxins15110664)

**Figure S1.** Statistical analysis of the data by season per year.

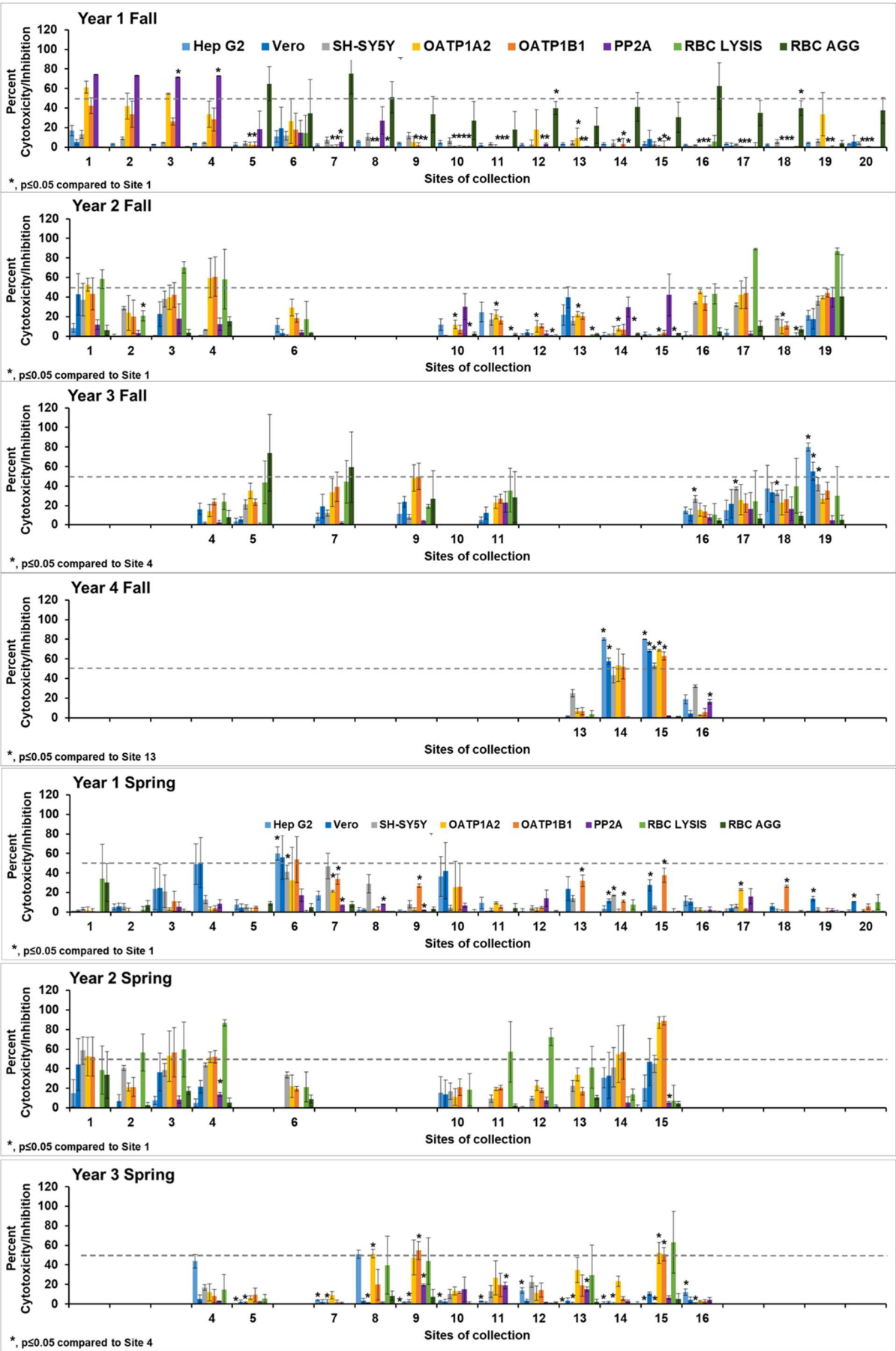

Supplement: Supplementary file 1 [file toxins-15-00664-s001.zip › Figure S1 toxins paper.pdf]
